# A Multiscale Approach for Whole-Slide Image Segmentation of five Tissue Classes in Urothelial Carcinoma Slides

**DOI:** 10.1177/1533033820946787

**Published:** 2020-10-14

**Authors:** Rune Wetteland, Kjersti Engan, Trygve Eftestøl, Vebjørn Kvikstad, Emiel A. M. Janssen

**Affiliations:** 1 Department of Electrical Engineering and Computer Science, 56627 University of Stavanger, Norway; 2 Department of Pathology, 60496 Stavanger University Hospital, Norway; 3 Department of Chemistry, Bioscience and Environmental Engineering, 56627 University of Stavanger, Norway

**Keywords:** bladder cancer, multiscale classification, tissue segmentation, deep learning, CNN, histological images

## Abstract

In pathology labs worldwide, we see an increasing number of tissue samples that need to be assessed without the same increase in the number of pathologists. Computational pathology, where digital scans of histological samples called whole-slide images (WSI) are processed by computational tools, can be of help for the pathologists and is gaining research interests. Most research effort has been given to classify slides as being cancerous or not, localization of cancerous regions, and to the “big-four” in cancer: breast, lung, prostate, and bowel. Urothelial carcinoma, the most common form of bladder cancer, is expensive to follow up due to a high risk of recurrence, and grading systems have a high degree of inter- and intra-observer variability. The tissue samples of urothelial carcinoma contain a mixture of damaged tissue, blood, stroma, muscle, and urothelium, where it is mainly muscle and urothelium that is diagnostically relevant. A coarse segmentation of these tissue types would be useful to i) guide pathologists to the diagnostic relevant areas of the WSI, and ii) use as input in a computer-aided diagnostic (CAD) system. However, little work has been done on segmenting tissue types in WSIs, and on computational pathology for urothelial carcinoma in particular. In this work, we are using convolutional neural networks (CNN) for multiscale tile-wise classification and coarse segmentation, including both context and detail, by using three magnification levels: 25x, 100x, and 400x. 28 models were trained on weakly labeled data from 32 WSIs, where the best model got an F1-score of 96.5% across six classes. The multiscale models were consistently better than the single-scale models, demonstrating the benefit of combining multiple scales. No tissue-class ground-truth for complete WSIs exist, but the best models were used to segment seven unseen WSIs where the results were manually inspected by a pathologist and are considered as very promising.

## Introduction

Worldwide, 549 393 new cases of bladder cancer were diagnosed in 2018, in addition there were 199 922 deaths due to the disease. This makes bladder cancer the 10th most common type of cancer in the world.^1^ Men are overrepresented, with approximately 75% of the cases.^2^ The most common type of bladder cancer is urothelial carcinoma, with over 90% of the cases.^3^ Of the patients diagnosed with bladder cancer, 50% to 70% will experience recurrence, and 10% to 30% will advance to a higher disease stage.^4^

Treatment and follow up of urothelial carcinoma are primarily based upon histological grade and stage, evaluated manually by an expert pathologist studying the histological images of the tumor using the latest WHO16 classification system.^5^ Correct grade and stage are essential to avoid over- or under-treatment, and thereby unnecessary suffering for the patient. For most pathology departments, evaluation of histological images is still performed through a microscope, a time-consuming process, not always reproducible.^6^ Digital pathology has been introduced to improve diagnostic accuracy, and certain computer-aided diagnostic (CAD) tools are in use for other diseases. However, such tools are currently not in use for the assessment of urothelial carcinoma and could potentially be of great value to patients and clinicians.

Non-muscle invasive bladder cancer is usually treated with transurethral resection of the tumor. The removed tissue contains both atypical urothelium from the tumor as well as stroma, but can also contain smooth muscle from the bladder wall, normal urothelium from surrounding mucosa and blood. During the procedure, parts of the tissue can get damaged, for example in terms of heating damage induced by laser or electrically heated wire loop. Areas on the whole-slide images (WSI) with blood and damaged tissue will not be suitable for extracting diagnostic and prognostic information, and a pathologist will discard such regions on inspection. CAD systems processing WSI must be able to identify trustworthy interesting areas of resected tissue, but also identify damaged areas and regions that should be excluded from further analyses.

**Figure 1.**
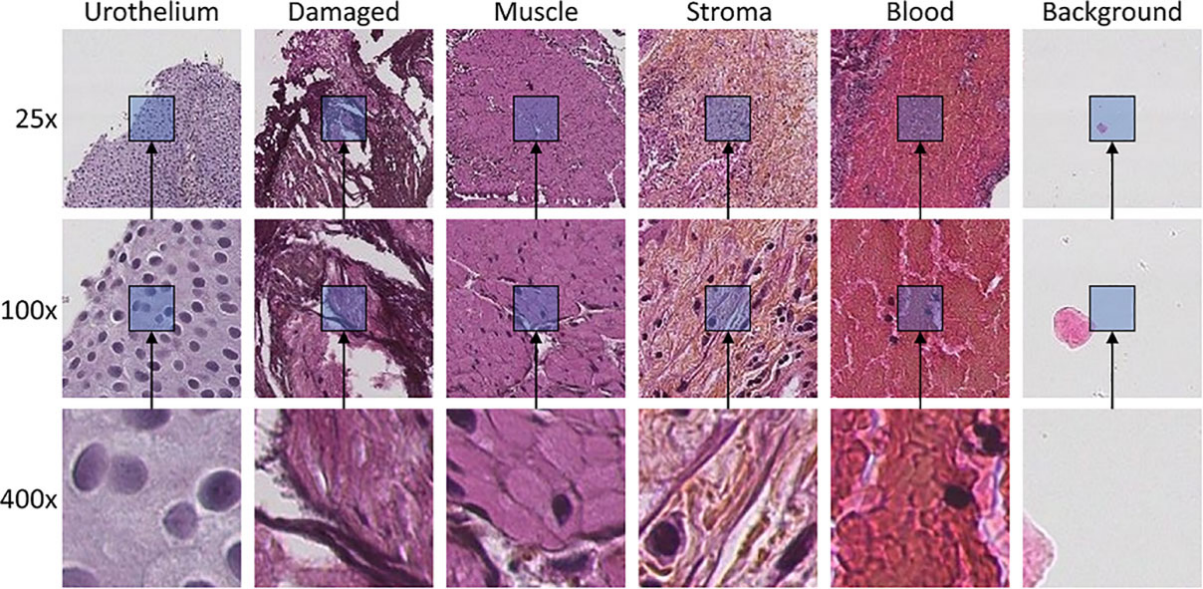
Example tiles of each class extracted at three magnification scales. Tiles at each scale are extracted from the same center pixel. The magnification scale is increased by a factor of 4 in each step, resulting in the tile covering 16 times as much area, even though they have the same size of 128 × 128 pixels.

This paper proposes an automatic method for classifying WSI tiles from urothelial carcinoma cases into the following categories: urothelium, stroma, muscle, damaged tissue, blood, and background, utilizing different magnification scales. Examples from each class are shown in Figure 1. The output of such a system can be used as a guide for pathologists, providing a quick visualization of where the different tissue types can be found. To the best of the author’s knowledge, a system for segmenting urothelial carcinoma WSIs into each tissue class does not exist. For determination of stage, pathologist wants to identify if muscle tissue is present or absent in the WSI and whether the tumor has infiltrated it. As muscle tissue is often sparse in the WSI, it can be time-consuming to get a full overview of its locations. However, with the help of segmented tissue images, it can be verified in a short amount of time. In the future, training data for a CAD system will be created by utilizing the best model developed through this paper by extracting diagnostic relevant features from the appropriate and relevant regions in the WSI. As this problem is not strictly dependent on classifying all six tissue classes, a binary approach is also experimented with in this paper classifying only urothelium vs. non-urothelium tissue to see if an increase in urothelium extraction can be achieved.

Tile-based classification of WSI has been done earlier.^7^ However, by only classifying a single tile, it leaves out information from the surrounding area. Moreover, WSI viewed on different magnification scale identifies different information. During an examination, a pathologist will integrate information across several magnification levels before reaching a final decision. Low magnification (25x) will show global context information such as papillary architecture, outline, and the border of the tissue, as well as color and texture. Nuclear polarity can be evaluated in the mid magnification (100x), while high magnification (400x) will reveal cytological features like cell size and shape, mitosis, as well as cell nucleus characteristics as contour, size and colorization intensity, and distribution.

The proposed method combines global context information found at lower magnifications (25x, 100x) with local information found at the highest magnification (400x) using deep neural networks to extract features from the different scales, thereafter concatenating the features feeding the last classifier layers of the network. Different neural network models were tested which utilized different combinations of the scales.

### Related Work

It is not possible to feed an entire gigapixel WSI into a deep neural network, and a practical solution to this is to divide WSI into tiles and feed the tiles sequentially to the deep neural network. There are primarily two methods for semantic segmentation within medical applications. The first, which utilizes models capable of providing pixel-wise classifications, can output segmentations with high resolution. These networks are usually based on the fully convolutional networks (FCN) introduced by Long et al. in.^8^ Popular models are the U-net model by Ronneberger,^9^ and variants of this.^10,11^ As these networks can detect small details, they are often used in cell and nuclei segmentation,^12,13^ but also on tumor segmentation tasks.^14^ The downside, however, is the need for pixel-wise ground-truth annotation for supervised learning, which is difficult and time-consuming to generate, especially in many medical applications. These networks are typically trained and tested on small example-patches from WSIs, since no dataset with a pixel-wise annotation of cells and tissue types on full WSI exist.

The second approach is based on tile-wise classification, where the models output a class label for each tile. This results in a coarser segmentation with the resolution of the tile size, and thus are more often seen for classification tasks rather than segmentation tasks. Nevertheless, it has been used in tumor segmentation methods.^15-19^ As every pixel within the tile belongs to the same class, the tile-based ground-truth annotation process is significantly simplified for classification and localization of regions within histological images.

A combination of both tile-wise and pixel-wise classification has been seen for segmentation of WSI by Guo et al.^20^ Firstly, a tile-based prediction using Inception-V3 gives a coarse segmentation of the WSI, followed by a pixel-wise classification of only the tumor tiles for refined segmentation of those areas. This approach can speed up the segmentation process relative to a pixel-wise segmentation of the entire slide; however, the need for pixel-wise ground-truth in all region of interests is still a significant challenge.

A pathologist studying a slide would typically zoom in and out, looking at both details and context. To similarly include these features in an artificial intelligence (AI) model, some multiscale approaches have been suggested. Models are trained with multiple input tiles, either taken from different magnification scales or taken from the same scale but with varying sizes to accommodate for a larger field of view. In the work of Sirinukunwattana et al.,^21^ the author has performed a systematic comparison between five single-scale and five multiscale architectures, tested on four classes of prostate cancer and four classes of breast cancer. Both tiles extracted at different magnification levels, as well as tiles of various sizes, were tested; and the result supports the claim that incorporating a broader visual context improves the outcomes. Another multiscale approach was used by Vu et al.,^13^ which created a network named multiscale deep residual aggregation network (MDRAN). First, a tile is extracted from the WSI at 200x magnification, and then resized to x0.5 and x2 the original size. The three scales (0.5x, 1x, 2x) were then aggregated in the model and used to accurately segment nuclei of non-small cell lung cancer (NSCLC). Since the models uses multiple inputs, the architectures often become more complex, and the total number of parameters within the models also goes up. This affects both the training and inference time of the models.

Most previous work on WSI classification is targeted on segmenting cancerous vs. non-cancerous areas of the WSI, and often the non-cancerous class may include several tissue classes. E.g. the work just mentioned by Vu et al.^13^ also performed WSI classification of NSCLC into three classes: NSCLC adeno (LUAD), NSCLC squamous cell (LUSC) and non-diagnostic (ND). The ND regions, in this case, consisted of fat, lymphocytes, blood vessels, red blood cells, normal stroma, cartilage, and necrosis without any attempt to separate these classes. Sometimes, however, there can be useful information in stroma, muscle, or other non-cancerous tissue types as well. There are some very few reported works on segmenting various tissue types. In,^22^ Li et al. propose a model with dual inputs trained to segment WSI from the ICIAR2018 breast cancer dataset into normal, benign, situ, and invasive regions. Also, a transfer learning model with multiple inputs was explored by Wang et al.^23^ to segment histological images of inflammatory bowel disease (IBD) into the four categories: muscle regions, messy regions, messy + muscle regions and background. Kather et al.^24^ used a deep learning model to classify tiles from colorectal cancer into eight different classes of tissue: tumor epithelium, simple stroma, complex stroma, immune cell conglomerates, debris and mucus, mucosal glands, adipose tissue, and background.

Relatively little work is aimed at segmentation of bladder cancer WSIs. In the work of Xu et al.,^18^ a method for predicting low or high tumor mutational burden (TMB) in bladder cancer patients was investigated. As a preprocessing step, a tile-wise tumor vs. non-tumor classifier was used to segment out the tumor regions from the surrounding tissue. An SVM classifier was then used to predict the patient’s TMB state using extracted histological image features from the tumor regions. A similar approach was used by Zhang et al.,^14^ where a U-net like network was used to predict each pixel into tumor or non-tumor as a preprocessing step before using another neural network for predicting the slide level diagnosis. As urinary bladder tumors are removed using a laser, burnt and damaged tissue is often present at the WSI. Muscle, stroma, and blood will also be part of the removed tissue and visible in the WSIs. But no effort is aimed at identifying these regions, even though they may contain valuable information for a pathologist.

The recent research efforts show promising results utilizing deep neural networks in different configurations for classifying and localizing cancerous areas. However, most effort is made on the “big four” in cancer (i.e., breast, lung, prostate, and bowel), performed on some publicly available datasets. Still, there is relatively little work done on other cancer types, on multiclass classification, on tissue-type classification, and segmentation/heat maps of full WSI.

### Aims and Contributions

In Wetteland et al.,^25^ we presented a method based on convolutional neural networks (CNN) for classifying tiles of urothelial carcinoma WSI into the six classes shown in Figure 1. The model utilized the autoencoder architecture and was first pre-trained on a large unlabeled dataset, and afterward fine-tuned on an annotated dataset. The models did not include any context, as both the unlabeled and labeled dataset was extracted at the full image resolution of 400x magnification.

The main contribution of the current paper is to combine histological images from different magnification scales into the model, giving the model access to a greater field of view and more context of the surrounding tissue. The resulting models are also used to generate segmented images of all the tissue classes within bladder cancer WSIs. An extensive number of experiments are conducted to find the best combination of inputs and magnification levels for the given task. The method utilizes the pyramidical image file format to extract tiles from existing down-sampled versions already present in the file, excluding any up- or down-sampling, limiting the number of necessary computational operations. Transfer learning is incorporated by building on the VGG16 network rather than the autoencoder model. To summarize, this paper proposes an automatic multiscale system, merging inputs of 25x, 100x, and 400x magnification, based on a CNN for classification of whole-slide histological images into six classes.

A preliminary study of this work was published by Wetteland et al. as an abstract.^26^ Here we present much more comprehensive experimental work and a description of the method.

## Materials and Methods

First, the data material will be introduced and explain how the datasets are prepared. Afterward, the proposed system for tissue segmentation is presented. Then the structure of the model is described, and finally, the training procedure and model selection is explained.

### Data Material

The data material consists of digital whole-slide images from patients diagnosed with primary papillary urothelial carcinoma, collected at the University Hospital of Stavanger, Norway, in the period 2002-2011. The biopsies are formalin-fixed and paraffin-embedded, from which 4 μm slices are cut and stained with Hematoxylin Eosin Saffron (HES).

**Figure 2.**
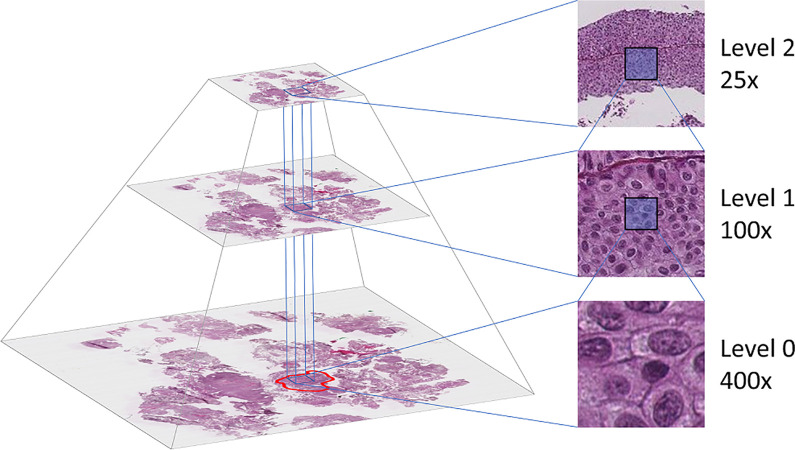
The WSI is stored in a pyramidal file format, including several down-sampled versions of the base image. The annotated region (marked with red at level 0) determines which tiles to extract. Tiles are then extracted at the desired location from all three levels.

The prepared tissue samples are scanned at 400x magnification using the Leica SCN400 slide scanner, producing image files in Leica’s SCN file format. The images are stored as a pyramidal tiled image with several down-sampled versions of the base image in the same file to accommodate for rapid zooming. Each level in the file is down-sampled by a factor of 4 from the previous level. Figure 2 shows an example of a pyramidal histological image with three levels. The Vips library^27^ is capable of extracting the base image as well as the down-sampled versions, making it easy to extract the dataset at each resolution.

Two datasets were collected from the described data material, referred to as the CV dataset and the inference dataset, both are described below.

#### CV dataset

An expert pathologist carefully annotated selected regions in the WSI, where each region includes one of the six classes. A total of 239 regions belonging to the five foreground classes was annotated in WSI from 32 unique patients. The background regions were extracted from seven randomly selected patients.

The annotated regions contain tight corners and narrow passages to accommodate the shape of the tissue regions in the WSI. When extracting tiles from the WSI, a grid of non-overlapping tiles was superimposed upon the annotated region at 400x magnification level. The tiles in the grid which lie outside of the region are regarded as invalid and will not be used, whereas tiles within the region are valid. By shifting the grid in the X- and Y- direction, more or fewer tiles become valid. To maximize the number of valid tiles, an automatic search algorithm was developed. The algorithm checks the number of valid tiles for all possible positions of the grid. The grid location with the highest number of valid tiles was used to extract the dataset from that region. This search was performed individually for each region.

Tile sizes of 64 × 64, 128 × 128, and 256 × 256 pixels were tested when extracting tiles with the automatic program. Using a tile size of 64 × 64 extracted the most extensive dataset, but the size may be too small as each tile contain little context information. With a tile size of 256 × 256, the extracted dataset became very small, especially for the stroma and muscle class. A tile size of 128 × 128 was thus chosen as a trade-off between the other two sizes. When a tile is saved from the region, the corresponding tiles from 25x and 100x magnification were also extracted in such a manner that the center pixel is the same in all three magnification levels, as can be seen in the right-half of Figure 2.

The extracted 400x magnification tiles are ensured to stay within the region border. However, by keeping the tile size the same, the lower magnification (25x, 100x) tiles will have a wider field of view, allowing for more context of the surrounding tissue to be included. Consequently, these tiles will, in some cases, include several classes. Because the annotation process requires specific expertise input, the dataset contains a limited number of samples. Furthermore, the labels are imprecise as they do not include samples of the labeled border between tissue regions. This would require multi-label samples, an even more expensive annotation process. As a result of this, the dataset is weakly labeled in both quantity and quality.

No normalization of the stain color is performed on the data, and the raw pixel intensity is used to train the models.

Stroma- and muscle-tissue are more sparsely distributed in the WSI, resulting in a smaller amount of data for these classes. Data augmentation techniques have been utilized to balance the dataset. Tiles from these two classes are extracted with 50% overlap, and further rotated and flipped during training to achieve a more balanced dataset. The size of each class is listed in Table 1.

**Table 1.** The Resulting CV Dataset Is Listed in the Table With the Total Number of Tiles Extracted for Each Class. The Number of Tiles Refers Only to Tiles Extracted at 400x Magnification. For the DI- and TRI-CNN Models, the Numbers Need to be Multiplied by 2 and 3, Respectively. Classes Marked With an Asterisk Shows the Number of Tiles After Augmentation.

Class	Tiles	Patients
Urothelium	29 728	28
Damaged	33 607	9
Stroma*	9 750	5
Blood	19 832	5
Muscle*	19 932	4
Background	27 012	7

Due to the low number of patients in the dataset, a traditional train/validation/test split could potentially hurt both the training and evaluation of the models. Instead, stratified 5-fold cross-validation is used. This enables the usage of all WSIs in both training and testing of the models. Stratification is performed on the patient-level to ensure that tiles from the same patient are not present in both the training and test set. A random seed is set to ensure that the folds are the same for each model, making the included samples in the training and test sets identical for all models.

#### Inference dataset

In addition to the CV dataset, seven WSIs were selected to be used as inference on the retrained models. The WSIs included in the inference dataset is not part of the CV dataset, and thus unseen by the models. As with the CV dataset, no normalization is performed on the WSIs in the inference dataset.

Due to the large size of the histological images, the WSIs included in the inference dataset do not have any annotations, and therefore any quantitative measurements are lacking. However, the resulting segmented images have been examined by a pathologist to be promising and confirm that the models can go from predicting smaller regions of the WSI to segment the full WSI.

### Proposed System

**Figure 3.**
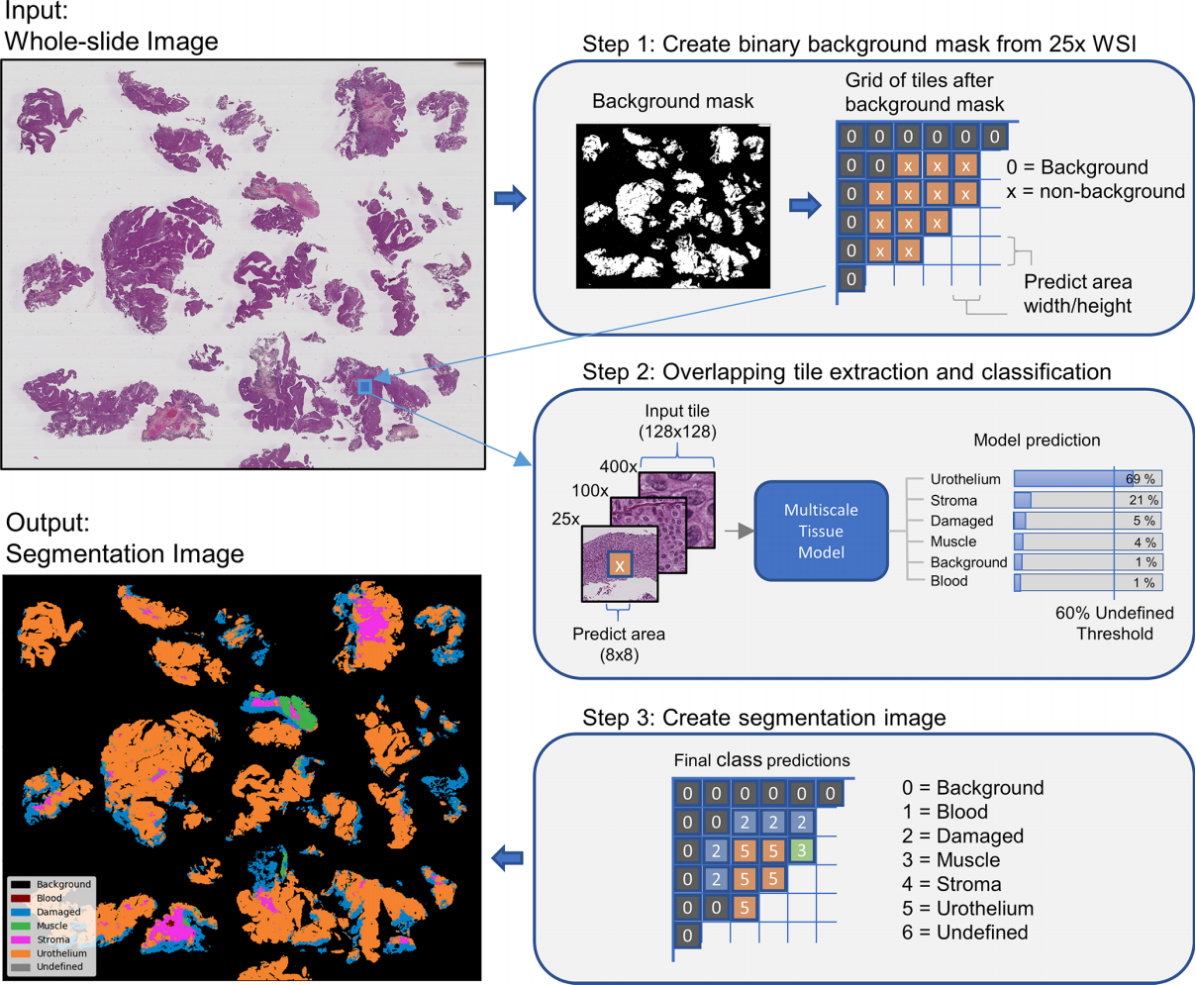
Overview of the proposed system. A background mask is created from the 25x WSI to exclude the background from further processing. Areas in the WSI selected as non-background is then extracted and fed through the multiscale model from Figure 4, which outputs tissue predictions. The prediction needs to exceed a set threshold to be valid. Finally, the segmentation image is generated by giving each class a separate color. The values shown in the figure are for illustration purposes only.

An overview of the proposed system for tissue segmentation of whole slide images is presented in Figure 3. The system accepts input WSI of any size and outputs a corresponding segmentation image from the input. The system is tested on the seven WSIs in the inference dataset. The system consists of three main steps which will be described here. The multiscale model in step 2 is described in more detail in the next section. Note that the blue box in step 2 in Figure 3 marked with “Multiscale Tissue Model” can be exchanged with any of the models described in the model structure section below.

**Figure 4.**
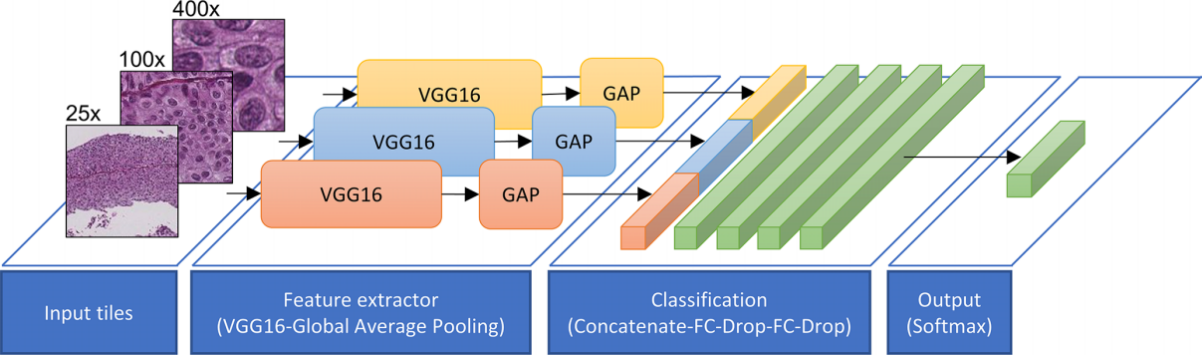
A block diagram of the TRI-CNN model proposed in the current paper. The input tiles are fed through individual pre-trained VGG16 network and global average pooling (GAP) layer to create feature vectors. The feature vectors are concatenated and fed through the classification network before entering the final output layer consisting of a softmax function. The softmax function outputs a prediction score for each of the six classes.

First, a binary background mask is produced from the 25x level of the WSI, generated by checking the pixel intensity value and splitting them into background or non-background tiles. About 60 to 80% of the WSI is covered by background, so this step reduces the number of tiles that needs to be processed by the inference model. Tiles selected as non-background are then extracted and fed to the multiscale model for further classification.

Depending on which model architecture is used (MONO, DI, or TRI), one, two, or three tiles are extracted from the same location but with different magnification. The extracted tile will always be 128 × 128 pixels, as this is the required input size of the inference model. However, the prediction only holds for a smaller area within the tile, typically 8 × 8 pixels, but can be set to any size. The input tiles are then overlapped, such that the inner area is located next to each other with no overlap.

Tiles are classified according to the highest prediction score. The outcome of a prediction may be equally split between multiple classes (e.g., two classes getting a score of 0.5 each, or four classes getting 0.25 each). To avoid such cases, a threshold value is set to determine if a prediction is valid. To ensure that the majority of the predicted score falls to a single class, the threshold needs to be above 0.51. Also, by setting the threshold too high may result in removing too many tiles. A threshold value of 0.6 is therefore determined as a trade-off between removing the unwanted conflicting predictions and not removing too much. Tiles with all prediction scores below the threshold are labeled as *undefined*.

Finally, each class is given a separate color, and the final segmentation image is saved. The segmentation images are ensured to only show classes with prediction scores higher than 0.6 but do not show the exact score. A method for creating heat maps has also been implemented, where no thresholding is performed, and the score for each class is visualized. A disadvantage of this is that one image must be created for each class. We earlier showed this approach in Wetteland et al.,^25^ but have omitted it from this paper.

#### Multiscale model structure

This paper compares three architectures referred to as the MONO-, DI-, and TRI-CNN models. The three architectures have one, two, and three inputs, respectively. To differentiate the models from each other, they are named according to their main architecture, and the input scale, e.g. MONO-400x is a MONO-CNN model trained on tiles extracted at 400x magnification. Tiles in the dataset are extracted at three magnification levels, yielding three MONO models: MONO-25x, MONO-100x, and MONO-400x. These three magnification scales can further be combined in three configurations for the DI-CNN model: DI-25x-100x, DI-25x-400x, and DI-100x-400x. The TRI-CNN model has only one configuration: TRI-25x-100x-400x, and is depicted in Figure 4. The different MONO- and DI-CNN models can easily be derived from the same figure. E.g. to create the DI-25x-400x model, remove the 100x input and blue blocks, and to create the MONO-100x model, remove the 25x input, 400x input, red and yellow blocks.

The overall structure of each model is the same. Each input is fixed at 128 × 128 × 3 pixels, which is the size of each tile. The input is fed into a pre-trained VGG16 network^28^ which acts as a feature extractor, followed by a global average pooling (GAP) layer providing a feature vector representation of the input. This feature vector is then fed into a classification network consisting of two fully-connected (FC) layers, each followed by a dropout layer, and a final softmax layer with one output node for each class. The DI- and TRI-CNN models have two and three parallel VGG16 branches, respectively, resulting in multiple feature vectors. These feature vectors are concatenated before entering the classification network. The FC-layers has the same size of 4096 neurons as the original layers in the VGG16 network. Dropout layers are added after each FC-layers to add regularization to the network due to the small dataset.

#### Training procedure and model selection

All models were trained using the SGD optimizer with a learning rate of 1.5e-4, batch size of 128, a dropout rate of 0.3, and a cross-entropy loss function. Early stopping was enabled, stopping the model when no increase in performance during the past 10 epochs was seen. Due to the cross-validation training scheme, no validation set was used, and the early stopping process was thus monitoring the training loss. The model is written in Python 3.5 using the Keras machine learning library,^29^ and Scikit-learn module^30^ for evaluation.

The models were trained in a stratified 5-fold cross-validation fashion. To produce an unbiased evaluation score, the output from each fold was summarized in a micro-average manner, as suggested by Forman and Scholz.^31^ All the true positive (TP), false positive (FP), and false negative (FN) values were summarized for each class over all the folds to produce a final micro-averaged F1-score.

The VGG16 network, which is used as a base model in our architectures, is pre-trained on the ImageNet dataset.^32^ It is possible to have the base model fixed during training by freezing the parameters, preventing the base model from being updated. Freezing the parameters will allow for faster training as fewer parameters need to be learned, however, as the nature of the histological images is not part of the ImageNet domain, it could affect the model’s ability to fully grasp the new images. By unfreezing the weights, it may allow to better adapt to the histological domain, at the cost of longer training time. Both freezing and unfreezing the weights were tested in the experiments.

As one of the objectives is to be able to automatically extract *urothelium tissue* from the histological images, to be used in diagnostic systems in the future, it is therefore not strictly necessary to classify all six tissue classes. A possible easier problem would be to define a binary problem, classifying urothelium vs. non-urothelium tissue. Each model was therefore also tested with this binary-class approach to see if it improved classification results for urothelium tissue. By simply combining the remaining five classes into one non-urothelium class, the dataset becomes heavily unbalanced toward the non-urothelium class. To counteract against this, augmentation using rotation and flipping was applied to balance out the dataset. By augmenting all the tiles from the muscle, stroma, and urothelium class 4x during training, the dataset became evenly distributed between the two classes urothelium and non-urothelium.

After evaluating the model using stratified cross-validation, a new and final inference model was trained by utilizing all available data as training data. The average number of epochs used during cross-validation was used when training the inference model. This inference model was then used to predict new WSIs from the inference dataset.

## Results

This section will present the results for the different models. A total of 28 models were trained using stratified 5-fold cross-validation, including *single-* and *multiscale*, and *binary-* and *multiclass* models. Each model was trained using weakly labeled data, with both frozen and unfrozen weights in the VGG16 network.

**Figure 5.**
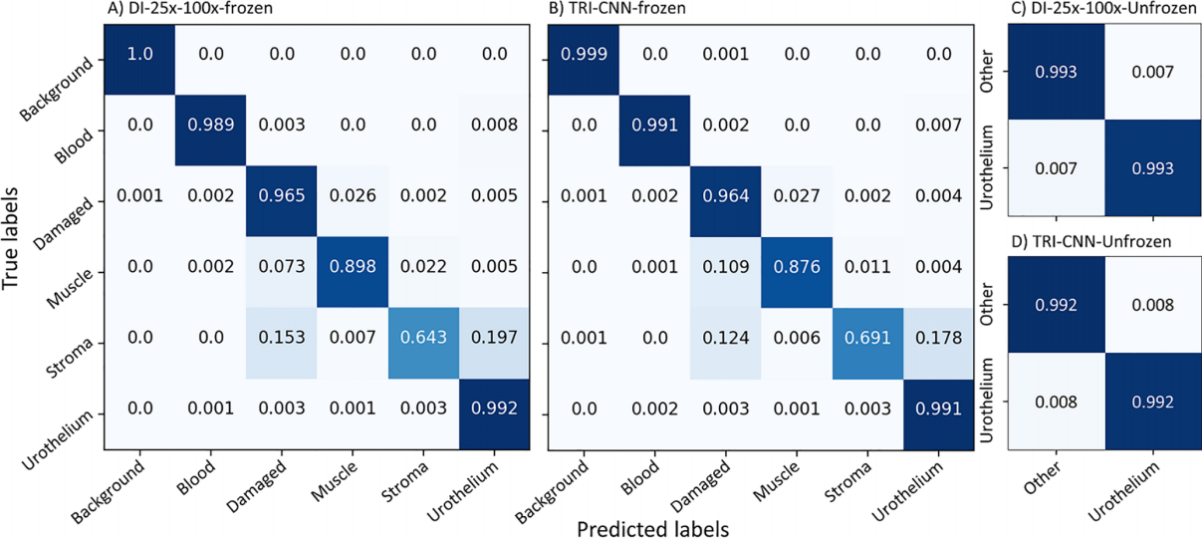
Normalized confusion matrices for the best multiscale models. Aggregated results across all 5 folds in the cross-validation test. A) Best multiclass DI-CNN, B) Best multiclass TRI-CNN, C) Best binary-class DI-CNN, and D) Best binary-class TRI-CNN.

Table 2 shows the cross-validation results for all the models. Aggregated micro-average F1-score across all classes are included, as well as the F1-score for only the urothelium class to better compare multiclass vs. binary-class models. Figure 5 displays the confusion matrices for the best multiclass models. The matrices are normalized to allow for more easy comparison. For the number of samples in each class, refer to Table 1.

**Table 2.** Results for all 28 Models, Trained Using Stratified 5-Fold Cross-Validation. Each Score Is Shown as Micro-Averaged F1-Score Aggregated Across all Classes, Marked as “All” in the Table. F1-Score Only for the Urothelium Class Is Shown in the Columns Marked “Uro.” Numbers in Bold Refer to the Highest Score in Their Respective Column.

		Multiclass				Binary-class			
		Frozen		Unfrozen		Frozen		Unfrozen	
	Model	All	Uro.	All	Uro.	All	Uro.	All	Uro.
Single-scale	MONO-25x	93.4	92.9	**96.4**	96.8	96.3	92.5	98.1	96.1
	MONO-100x	94.4	96.6	94.8	97.8	98.3	96.5	99.1	98.1
	MONO-400x	87.2	89.7	86.4	86.3	94.2	88.1	93.7	87.2
Multiscale	DI-25x-100x	**96.5**	97.4	96.2	98.1	98.1	96.2	**99.3**	**98.5**
	DI-25x-400x	95.6	96.3	96.0	97.6	97.8	95.4	98.3	96.5
	DI-100x-400x	95.0	96.8	95.3	97.6	98.4	96.6	98.9	97.7
	TRI-25x-100x-400x	**96.5**	**97.6**	**96.4**	**98.3**	**98.5**	**97.0**	99.2	98.3

**Figure 6.**
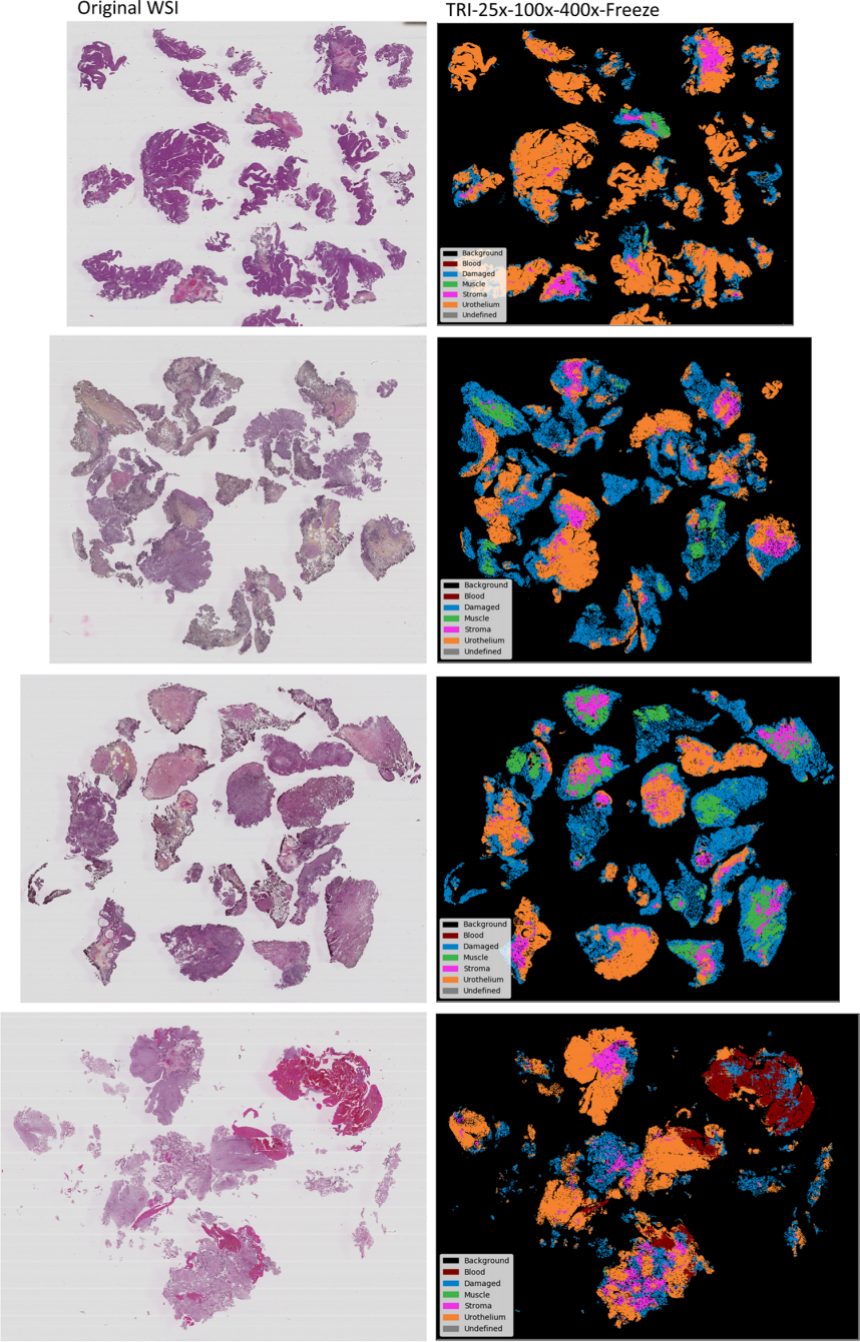
The best multiclass model was retrained and used to generate segmentation images from four WSI not present in the training data.

**Figure 7.**
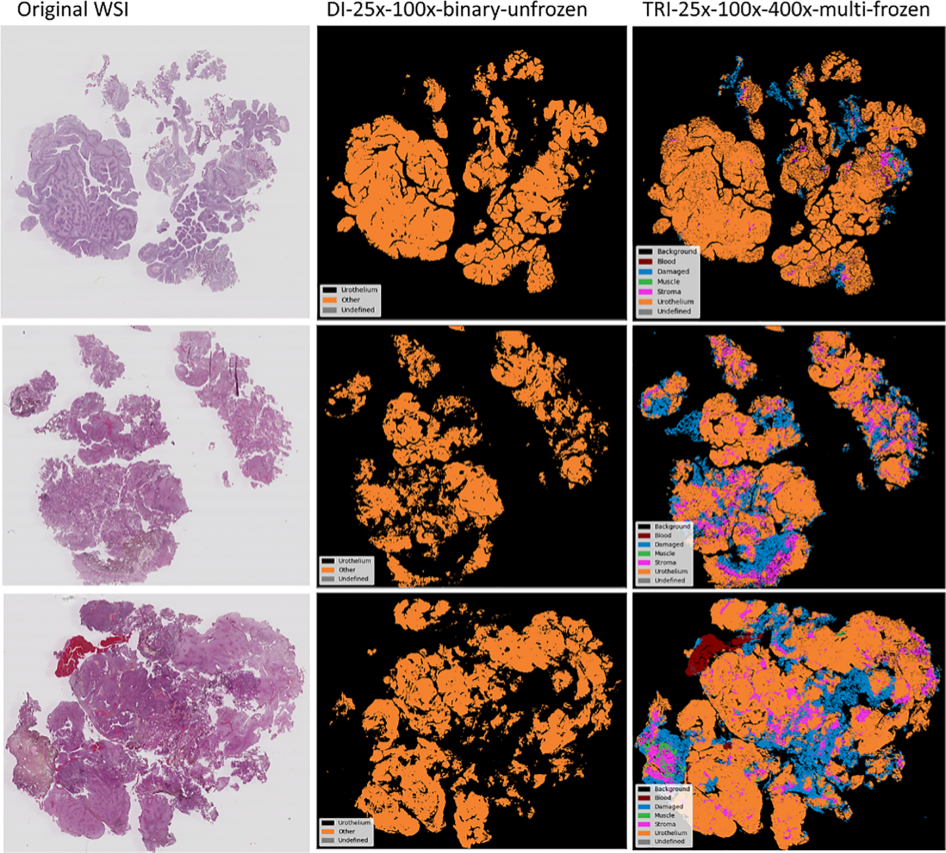
The best binary-class model vs. the best multiclass model. A DICE-score is calculated to measure the similarity between the predicted urothelium tissue between the two models. DICE-score from top to bottom are 0.92, 0.85 and 0.85 .

**Figure 8.**
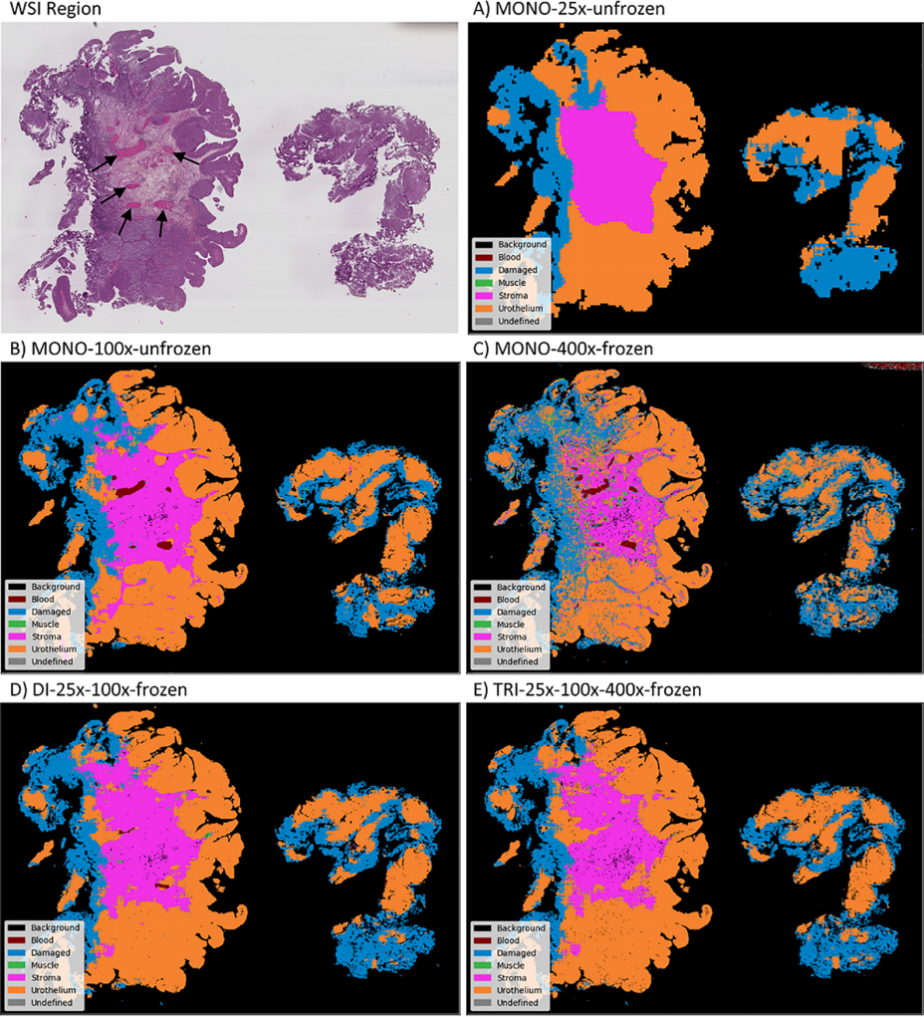
Segmentation of close-up region taken from the top-right corner from the first WSI in Figure 6. A) Best MONO-25x, B) Best MONO-100x, C) Best MONO-400x, D) Best DI-CNN model, E) Best TRI-CNN model. Arrows in the WSI region points to small areas of blood that the models struggle to identify.

Some of the best models have been retrained on the entire CV dataset and used to segment the seven WSIs included in the inference dataset. The resulting segmented images have then been inspected by an expert pathologist and are considered to be very promising. Figure 6 shows four WSIs and their corresponding tissue segmented images generated by the best multiclass model. Figure 7 shows a comparison between segmentation images generated by the best binary-class model and the best multiclass model. A DICE-score is calculated to measure the similarity between the predicted urothelium tissue between these two models, with an average DICE-score of 0.87 for the three WSIs. Figure 8 shows a close-up region taken from the top-right corner of the first WSI in Figure 6. This region is then segmented with all the best MONO-, DI-, and TRI-models for comparison.

## Discussion

The results in Table 2 are shown as micro-averaged F1-score across all classes, as well as for the urothelium class. The results are overall good for all models, and a discussion of each case follows below. Afterward, the confusion matrices and the segmented images will be discussed, and finally, different usage scenarios of the system will be considered as well as some limitations of the study.

### 

#### Binary-class vs. multiclass

As expected, the binary-class models achieve a higher average F1-score than the multiclass models, with all 14 of the binary models getting a higher score than their multiclass counterparts. This is expected because five of the classes are now grouped, and misclassification within these classes is canceled out. The best multiclass model is the frozen TRI-25x-100x-400x with an F1-score of 96.5% across six classes, whereas the best binary model is the DI-25x-100x with unfrozen weights, which got an F1-score of 99.3% across its two classes.

By looking at the F1-score for the urothelium class alone, the multiclass models are now superior, with 9 of the 14 models being ahead of their binary-class counterparts. The few binary-models which have a higher score, are only marginally so, with the largest difference being the unfrozen MONO-400x, where the binary version is 0.9% better than the multiclass version. It is clear that by simplifying the problem into a 2-class problem, did not help with getting better urothelium extraction. The highest urothelium score is achieved by the TRI model, where both the unfrozen multiclass and unfrozen binary-class version each got an equal F1-score of 98.3% for the urothelium class.

#### Frozen vs. unfrozen

The three architectures MONO, DI and TRI, have 19 M, 21 M, and 23 M trainable parameters, respectively, when the VGG16 weights are frozen. By unfreezing the weights, the same models get 34 M, 50 M, and 67 M trainable parameters. When comparing results for these models, there is on average an increase of +0.6% by unfreezing the weights. Of the 14 unfrozen models, 10 get a higher score than the corresponding frozen models. The largest increase is seen in the binary MONO-25x model, which goes from an F1-score of 96.3% to 98.1% by unfreezing the weights.

The increase in the number of trainable parameters also affects the training time of the models. The average time per epoch for all the frozen models was 9 minutes, while the unfrozen models needed on average 10 minutes to compute one epoch. This is an increase of 11% processing time per epoch. However, the frozen models needed on average 162 epochs to reach the early stopping criteria, whereas the unfrozen models only needed 58 epochs. Thus, the models with unfrozen weights needed about 60% less processing time during training.

#### Single-scale vs. multiscale

When comparing the single-scale MONO-models with the multiscale DI- and TRI-models, the multiscale models achieve better results across all columns in Table 2, with the exception for the unfrozen MONO-25x model which matches the performance of the TRI-scale model. If we limit ourselves to the multiclass models, the best models for the three architectures are the unfrozen MONO-25x with 96.4%, frozen DI-25x-100x with 96.5%, and frozen TRI-25x-100x-400x which got an F1-score of 96.5%. The story is similar for the binary models, with unfrozen MONO-100x being the best with 99.1%, unfrozen DI-25x-100x with 99.3%, and unfrozen TRI-25x-100x-400x with 99.2%.

By looking at the single-scale models alone, it is clear that the two lower scales (25x, 100x) are performing better than the 400x scale, and that having a greater field of view is preferable. The multiscale models, consisting of two and three VGG16 networks, have a more complex structure involving more parameters than the MONO models. In addition, they have access to a greater field of view in all its models. These two features seem to help the performance of these models.

Naturally, the MONO models take the least amount of training time, with an average of 4:40 minutes per epoch. The DI-models take 136% longer with an average of 11:01 minutes, and finally, the TRI-models take the most time with 19:38 minutes on average per epoch. That is 321% and 78% longer than MONO and DI, respectively. The average number of epochs before reaching the early stopping criterion for the three architectures was 147, 88, and 64 epochs for the MONO-, DI-, and TRI-models, respectively.

#### Confusion matrices

Figure 5 shows the resulting normalized confusion matrices for the best multiscale models for both multiclass and binary-class models.

In the two multiclass matrices (A) and (B), the models did an excellent job at classifying background, blood, and urothelium correctly, and a great job with the damaged class as well. Both models struggled mostly with the muscle and stroma classes. These are the classes with the fewest number of labeled samples in the dataset. As a result of this, the models may have achieved a weaker generalization for these classes, and thus misclassified them more often. Most notable misclassifications are related to muscle and stroma being misclassified as damaged tissue, and also stroma being misclassified as urothelium.

The two binary-class models in Figure 5 (C) and (D) got an equally good performance. Five of the classes are now combined into one class named *other* in the figure and thereby removing most of the misclassifications from the multiclass cases. However, this did not significantly increase the performance of model (C) and (D). Model (D) got the same normalized score as (A), and model (C) is only marginally better.

#### Inference dataset results

The seven WSIs included in the inference dataset were processed with overlapping tiles according to Figure 3, where only the inner 16 × 16 pixel of the tile was classified. The average processing time was 7 hours 18 minutes, including all three steps in Figure 3. On average, only 0.9% of the WSIs were categorized as undefined. Four of the WSIs are presented in Figure 6, and three in Figure 7.

#### Segmentation image results

The best multiclass model, according to Table 2, is split between two models. The frozen DI-25x-100x and frozen TRI-25x-100x-400x both have a similar F1-score of 96.5%, but the latter model has a higher urothelium F1-score and is thus regarded as the best multiclass model. The model was retrained and used to process four new WSIs, not present in the training data, to demonstrate its usage. Figure 6 shows the original WSI with the corresponding segmentation images. The segmented images are intuitive, easy to understand, and allow even untrained personnel to both identify and locate the difficult to find regions, e.g. like muscle tissue.

Fully multiclass-annotated WSI in our dataset is not available. The resulting segmentation images for the WSI have, however, been manually inspected by an expert uropathologist and are considered to be very promising, especially considering that the WSIs were only weakly annotated. Large homogeneous areas with a certain tissue type are clearly recognized. Most models are really challenged by smaller, more heterogeneous areas.

#### Binary-class vs. multiclass segmentation images

The best multiclass and binary-class models were retrained and used to create the segmentation images seen in Figure 7. The multiclass segmentation image may be of more interest to a pathologist, as it outlines regions of all six classes, whereas the binary-class segmentation image only outlines the urothelium class. However, both the multiclass and binary-class models have about the same F1-score for the urothelium class, and the additional information in the multiclass segmentation images favor the former model in a final system.

After comparing the urothelium regions in the two segmented images for each WSI, they are very similar. The DICE-score is calculated to measure the similarity between the regions, and the three cases have an average DICE-score of 0.87, which confirms that the two model’s prediction for urothelium is quite similar. However, there is no truth annotation, so the DICE-score does not reveal if one of the models is better than the other.

#### Close-up segmentation regions

Even though the system is trained on weakly labeled data, consisting of single-class samples, using tile-based classification and not a per-pixel classification, it is still interesting to see how the system performs on a detailed level. This also allows us to compare the different models. Figure 8 shows a close-up region taken from the top-right corner from the first WSI in Figure 6, processed using an 8 × 8 pixel predict area.

All models do a decent job of outlining the major regions in the image. The different models process the image on different scales, and so the prediction tile covers a larger area for the smaller scales. The effect of this is visible at the three MONO models, where the level of detail goes up with each scale. The MONO-100x and MONO-400x models, with its smaller field of view, are able to detect some of the small regions containing blood in the middle of the image. The MONO-25x, however, is not able to identify this. The DI-25x-100x model, which has access to both the mid and broad field of view, barely identifies a small part of the blood, whereas the TRI scale model does not identify it at all.

## Usage Scenarios

As seen from both Table 2 and the segmented images in Figure 6, the model is fully capable of distinguishing between the different tissue types. The presented system has several possible usage scenarios, which will be discussed here.

The segmented images in Figure 6 can be used as a digital tool for pathologists to help them become more efficient in their work. It can be used to guide them to the diagnostic relevant areas of the WSI, such as urothelium, muscle, and stroma tissue. It can also be used to find edges of the urothelium tissue without damage more easily. During an examination, a pathologist needs to verify if muscle tissue is present or not in the current WSI. With the segmented images, this can be verified within a short amount of time.

Another use case for the system is as a preprocessing step for an automatic diagnostic system. For instance, each patient has follow up records about whether the patient experienced recurrence and progression. By training a diagnostic model on the entire WSI, the dataset quickly becomes too large if many patients are included. Also, by randomly selecting a subset of tiles within each WSI, the dataset will include a large portion of damaged tissue and blood, which will add noise to the diagnostic model. By using the multiscale tissue model presented in this paper as a preprocessing step, areas of clean, undamaged urothelium and other diagnostic relevant types can easily be extracted and used as training data.

### Limitations

One limitation of the current study is that the dataset is relatively limited in size. A small training dataset may lead to overfitting of the model, resulting in poor performance, and a small test set may cause an optimistic estimate of the performance. Several measures have been taken to reduce these negative effects. Pre-trained models, dropout, and early stopping was used to reduce overfitting, and cross-validation was used to get a realistic estimate of each model’s performance.

As mentioned in the data material section, the labels are accurate in the highest resolution (400x) but are imprecise on the lower scales (25x, 100x), meaning the ground-truth is based on weak annotations of the dataset, which may impact the accuracy. The experimental results show that having access to a greater field of view outweighs the potential negative effects of imprecise labels.

It is difficult to compare the presented models against other approaches or to perform a test on an independent dataset. To the best of the authors’ knowledge, no other open dataset exists with annotations of the same six classes. As mentioned in the related work section, some research and models exist for segmentation of histological images. However, these are based on other cancer types or trained on other classes than the six classes used in this paper.

## Conclusion

This paper investigates the effect of using multiple scales during tissue classification from WSI of urothelial carcinoma into six classes. The classification is performed on smaller tiles and can be useful for a coarse segmentation, or ROI-extraction, of WSI. Three main architectures are presented: MONO-, DI-, and TRI-CNN model, and a total of 28 different models were trained using weakly labeled data and evaluated in a stratified 5-fold cross-validation scheme.

The multiscale models achieved a better result than the MONO-CNN models. There was not a substantial increase in urothelium classification by using the binary-class models, neither by cross-validation or by inspection of the segmented images. The best multiclass model was used to generate intuitive and easy to understand segmented images from unseen WSIs, and after inspection by a pathologist is considered to be very promising.

The segmented regions shown in Figure 8 demonstrates the importance of including the highest magnification scale (400x) during tile-wise classification. The models which do not include this scale are not able to identify the smaller details within the WSI.

As the three MONO models pick up different levels of details, we will in the future experiment on employing them in a multiscale ensemble model by combining their outputs, instead of combining the different scales within the models, as the DI- and TRI-CNN models do. We also plan to use the model for automatic ROI-extraction of relevant tissue in the WSI to create training datasets for a diagnostic and prognostic classification model. By only extracting the diagnostic relevant areas of the WSIs, a dataset of much higher quality can be collected.

## Abbreviations

AI: artificial intelligence
CAD: computer-aided diagnostic
CNN: convolutional neural network
CV: cross-validation; FC, fully-connected
FCN: fully convolutional networks
FN: false negative
FP: false positive
GAP: global average pooling
HES: Hematoxylin Eosin Saffron
IBD: inflammatory bowel disease
MDRAN: multiscale deep residual aggregation network
NSCLC: non-small cell lung cancer
ROI: region of interest
SGD: stochastic gradient descent
SVM: Support vector machine
TMB: tumor mutational burden
TP: true positive
WHO: World health organization
WSI: whole-slide image
